# Sparse Method for Direction of Arrival Estimation Using Denoised Fourth-Order Cumulants Vector

**DOI:** 10.3390/s18061815

**Published:** 2018-06-04

**Authors:** Yangyu Fan, Jianshu Wang, Rui Du, Guoyun Lv

**Affiliations:** School of Electronics and Information, Northwestern Polytechnical University, Xi’an 710129, China; fan_yangyu@nwpu.edu.cn (Y.F.); rdu@mail.nwpu.edu.cn (R.D.); lvguoyun101@nwpu.edu.cn (G.L.)

**Keywords:** direction of arrival estimation, fourth-order cumulants, non-Gaussian sources, fourth-order difference co-array, sparse Bayesian learning

## Abstract

Fourth-order cumulants (FOCs) vector-based direction of arrival (DOA) estimation methods of non-Gaussian sources may suffer from poor performance for limited snapshots or difficulty in setting parameters. In this paper, a novel FOCs vector-based sparse DOA estimation method is proposed. Firstly, by utilizing the concept of a fourth-order difference co-array (FODCA), an advanced FOCs vector denoising or dimension reduction procedure is presented for arbitrary array geometries. Then, a novel single measurement vector (SMV) model is established by the denoised FOCs vector, and efficiently solved by an off-grid sparse Bayesian inference (OGSBI) method. The estimation errors of FOCs are integrated in the SMV model, and are approximately estimated in a simple way. A necessary condition regarding the number of identifiable sources of our method is presented that, in order to uniquely identify all sources, the number of sources *K* must fulfill $K\leq (M^{4}-2M^{3}+7M^{2}-6M)/8$. The proposed method suits any geometry, does not need prior knowledge of the number of sources, is insensitive to associated parameters, and has maximum identifiability $O(M^{4})$, where *M* is the number of sensors in the array. Numerical simulations illustrate the superior performance of the proposed method.

## 1. Introduction

Direction of arrival (DOA) estimation is a topic that has received a great deal of attention in the last several decades. It arises in many practical scenarios, such as radar, sonar, and communications [1,2,3,4]. For example, in radar and sonar systems, objects including airplanes, birds, missiles, submarines, and fish torpedoes can be tracked by estimating their DOAs. In communication systems, the DOAs of the sources can be used to improve the communication quality.

Higher-order cumulants (HOCs)-based DOA estimation methods have been developed for non-Gaussian sources [1,5,6,7,8], mainly to overcome the limitations of second-order statistics-based DOA estimation methods such as MUltiple SIgnal Classification(MUSIC) [9] and Estimation of Signal Parameters via Rotational Invariance Techniques (ESPRIT) [10]. Their limitations include identifiable number of sources, colored Gaussian noise, and modeling errors. The famous 4-MUSIC method [5] uses the fourth-order cumulants (FOCs) to form a MUSIC-like method. Then, the 2q-MUSIC [6] and rectangular 2q-MUSIC [7] methods extend 4-MUSIC by utilizing 2qth-order cumulants. The rectangular 2q-MUSIC method achieves a trade-off between performance and maximal identifiable number of sources compared with the 2*q*-MUSIC method. To further increase the degrees of freedom (DOFs) of the FOCs-based methods, a kind of sparse linear array named multiple level nested array and the corresponding spatial smoothing MUSIC (SS-MUSIC) method were developed, which make the DOFs increase from $O(M^{q})$ to $O(M^{2q})$ [8], where *M* is the number of sensors in the array. However, HOCs-based methods always suffer from performance degradation when snapshots are limited, mainly because accurate estimations of HOCs require a large number of snapshots.

Recently, a series of covariance vector-based DOA methods were proposed [11,12,13] which built a single measurement vector (SMV) model by covariance vector and were solved by sparse methods such as L1-norm-based methods [14] and sparse Bayesian learning (SBL) [15]. Consequently, these methods always perform better than other covariance-based DOA methods such as MUSIC when snapshots are limited, mainly because estimation errors of covariance are utilized to build their sparse models. Moreover, the DOFs of these methods increase to $O(M^{2})$, which is more than MUSIC. Analogously, the FOCs vector-based L1-norm method (4-L1) is applied in [16,17,18]. Compared with FOCs vector-based SS-MUSIC (4-SS-MUSIC) in [8], the 4-L1 method can utilize all the virtual sensors in the fourth-order difference co-array (FODCA) [8], which may give a better performance than 4-SS-MUSIC. Moreover, the 4-L1 method can be applied to any array, while 4-SS-MUSIC only suits for linear arrays. Nonetheless, the 4-L1 method is inconvenient to use in practice, since the parameter of allowable error bound is difficult to choose, and the solution of 4-L1 is sensitive to it. The allowable error bound usually needs to be chosen manually through trial-and-error for each scenario, as in [16,17,18]. Furthermore, it has been demonstrated both theoretically and empirically that the SBL technique induces less structural error (biased global minimum) and convergence error (failure in achieving the global minimum) than the L1 methods [11,19,20,21]. On the other hand, an advanced denoising procedure of the FOCs vector may further promote the performance of FOCs vector-based DOA estimation methods. An analogous procedure can be found in covariance vector-based DOA methods in [22].

In this paper, we establish a novel denoised SMV model by FOCs vector, and solve it by an off-grid sparse Bayesian inference (OGSBI) method [21]. By utilizing the concept of the fourth-order difference co-array, the advanced denoising or dimension reduction procedure of FOCs vector is presented for any geometry. The estimation errors of FOCs are integrated in the proposed SMV model, and are approximately estimated in a simple way. A necessary condition of our method regarding the number of identifiable sources is presented such that in order to uniquely identify all sources, the number of sources *K* must fulfill $K\leq (M^{4}-2M^{3}+7M^{2}-6M)/8$. The proposed method suits for any geometry, does not require prior knowledge of the number of sources, has maximum identifiability $O(M^{4})$, and is insensitive to associated parameters. The off-grid parameter estimation in our method promotes superior performance in overdetermined cases when the number of snapshots is large and signal–noise-ratio (SNR) is high, and also ensures good performance when choosing a relatively coarse grid. Numerical simulations illustrate the superior performance of the proposed method.

## 2. Data Model

One-dimensional DOA estimation is discussed in this paper. Consider an array with any geometry which consists of *M* identical, omnidirectional, unpolarized and isotropic sensors. The sensor locations $n_{1},n_{2},\ldots ,n_{M}$ are measured by d=λ/2, and collected by the vector set $S≜\left\{n_{1},n_{2},\ldots ,n_{M}\right\}\subset R^{3}$, where $n_{m}={\left[n_{m,x},n_{m,y},n_{m,z}\right]}^{T}$ is expressed in three dimensions for m=1,2,…,M, ${\left(\cdot \right)}^{T}$ is the transpose operator, λ is the wavelength, and R denotes the set of real numbers. The cardinality of S is denoted by |S|, and |S|=M. Each sensor receives the contribution of *K* zero-mean statistically independent stationary narrowband non-Gaussian sources. We denote by $x\left(t\right)={\left[x_{1}\left(t\right),x_{2}\left(t\right),\ldots ,x_{M}\left(t\right)\right]}^{T}$, $s\left(t\right)={\left[s_{1}\left(t\right),s_{2}\left(t\right),\ldots ,s_{K}\left(t\right)\right]}^{T}$ and $v\left(t\right)={\left[v_{1}\left(t\right),v_{2}\left(t\right),\ldots ,v_{M}\left(t\right)\right]}^{T}$ the observed signals of the sensors, the source signals, and the noises of the observed signals at the *t*th snapshot, respectively, where t=1,2,…,+∞. The observed signals are modeled as:

(1) $$\begin{array}{c} x(t)=\mathrm{As}(t)+v(t), \end{array}$$

where $A=\left[a\left(\theta_{1}\right),a\left(\theta_{2}\right),\ldots ,a\left(\theta_{K}\right)\right]\in C^{M\times K}$ denotes the array manifold matrix, $a\left(\theta_{k}\right)={\left[e^{j2\pi u_{k}^{T}n_{1}},e^{j2\pi u_{k}^{T}n_{2}},\ldots ,e^{j2\pi u_{k}^{T}n_{M}}\right]}^{T}$ is the steering vector for $\theta_{k}$, j is an imaginary number, $u_{k}=d/\lambda \cdot {\left[\cos\left(\theta_{k}\right),\sin\left(\theta_{k}\right),0\right]}^{T}$, $\theta_{k}$ denotes the azimuth angle of the *k*th source, and C is the set of complex numbers. The noises are assumed to be statistically independent zero-mean white Gaussian and independent from each source.

Build the FOCs vector $c_{x}\in C^{M^{4}}$, where the *i*th entry is ${\left[c_{x}\right]}_{i}=\mathrm{cum}\left\{x_{i_{1}}\left(t\right),x_{i_{2}}\left(t\right),x_{i_{3}}^{*}\left(t\right),x_{i_{4}}^{*}\left(t\right)\right\}$ with $i=\left(i_{1}-1\right)M^{3}+\left(i_{2}-1\right)M^{2}+\left(i_{3}-1\right)M+i_{4}$, cum{·} denotes the cumulant operator, ${\left(\cdot \right)}^{*}$ denotes complex conjugate, and the indexes $i_{\gamma }$ and γ are integers satisfying $1\leq i_{\gamma }\leq M$ and 1≤γ≤4, respectively. Due to model (1), the above assumptions of the sources and noises, and the properties of the cumulants [23,24], we can obtain

(2) $$\begin{array}{c} {\left[c_{x}\right]}_{i}=\sum_{k=1}^{K}c_{s_{k}}a_{i_{1}}\left(\theta_{k}\right)a_{i_{2}}\left(\theta_{k}\right)a_{i_{3}}^{*}\left(\theta_{k}\right)a_{i_{4}}^{*}\left(\theta_{k}\right), \end{array}$$

where $c_{s_{k}}=\mathrm{cum}\left\{s_{k_{1}}\left(t\right),s_{k_{2}}\left(t\right),s_{k_{3}}^{*}\left(t\right),s_{k_{4}}^{*}\left(t\right)\right\}$ with $k_{\gamma }=k$, $a_{i_{\gamma }}\left(\theta_{k}\right)$ is the $i_{\gamma }$th element of $a_{\gamma }\left(\theta_{k}\right)$, and $a_{\gamma }\left(\theta_{k}\right)=a\left(\theta_{k}\right)$. Then, we have

(3) $$\begin{array}{c} c_{x}={\mathrm{Bc}}_{s}\in C^{M^{4}}, \end{array}$$

where $c_{s}={\left[c_{s_{1}},c_{s_{2}},\ldots ,c_{s_{K}}\right]}^{T}$, $B=[b\left(\theta_{1}\right),b\left(\theta_{2}\right),\ldots ,b\left(\theta_{K}\right)]$, $b\left(\theta_{k}\right)=a_{1}\left(\theta_{k}\right)⊗a_{2}\left(\theta_{k}\right)⊗a_{3}^{*}\left(\theta_{k}\right)⊗a_{4}^{*}\left(\theta_{k}\right)$, and ⊗ denotes the Kronecker product.

## 3. The Proposed Method

In this section, we present an FOCs vector-based method to perform DOA estimation. Firstly, we provide a dimension reduction method to build a novel data model by employing the concept of a fourth-order difference co-array [8]. This method can also be treated as a denoising procedure to reduce the estimation errors of FOCs. Next, according to the newly built data model, our sparse model is efficiently established and solved by the OGSBI method. Some information about the proposed method (e.g., identifiability, complexity, and comparison with the state-of-the-art methods) is also discussed.

### 3.1. Dimension Reduction or Denoising Procedure

In this subsection, the dimension of model (3) is reduced by utilizing a fourth-order difference co-array. Here we supplement several definitions and properties to facilitate dimension reduction.

**Definition** **1.** *Fourth-order difference co-array (FODCA): Given an array with arbitrary geometry S, the fourth-order difference co-array D is defined as the set of distinct vector elements from the set*

(4) $$\begin{array}{c} D=\{n_{i_{1}}+n_{i_{2}}-n_{i_{3}}-n_{i_{4}}:\forall n_{i_{1}},n_{i_{2}},n_{i_{3}},n_{i_{4}}\in S\}, \end{array}$$

*where $i_{1},i_{2},i_{3},i_{4}=1,2,\ldots ,M$, as described in Section 2.*

Let $m_{1},m_{2},\ldots ,m_{\left|D\right|}$ denote the distinct elements in D, where |D| is the cardinality of D. The following definition gives the concept of a transformation matrix, which describes the relation between b(θ) and the steering vector on D (see Property 2 in this subsection).

**Definition** **2.** *The transformation matrix J: Given array S, the associated FODCA D can be obtained by Definition 1. The transformation matrix J is an $M^{4}\times \left|D\right|$ matrix, where the ξth column corresponds to the vector $m_{\xi }\in D$, and the (i,ξ)th entry of J satisfies*

(5) $$\begin{array}{c} J_{i,\xi }=\{\begin{array}{c} 1,\;\;\mathrm{if}\;n_{i_{1}}+n_{i_{2}}-n_{i_{3}}-n_{i_{4}}=m_{\xi },\\ 0,\;\;\mathrm{otherwise}, \end{array} \end{array}$$

*where the index ξ=1,2,…,|D|, and recall that $i=\left(i_{1}-1\right)M^{3}+\left(i_{2}-1\right)M^{2}+\left(i_{3}-1\right)M+i_{4}$ in Section 2.*

**Property** **1.** *The transformation matrix J has full column rank.*

**Property** **2.** *Let $a_{D}\left(\theta \right)$ be the steering vector of D (i.e., $a_{D}\left(\theta \right)={\left[e^{j2\pi u^{T}m_{1}},e^{j2\pi u_{k}^{T}m_{2}},\ldots ,e^{j2\pi u_{k}^{T}m_{\left|D\right|}}\right]}^{T}$), we have*

(6) $$\begin{array}{c} b\left(\theta \right)={\mathrm{Ja}}_{D}\left(\theta \right), \end{array}$$

where $b\left(\theta \right)=a_{1}\left(\theta \right)⊗a_{2}\left(\theta \right)⊗a_{3}^{*}\left(\theta \right)⊗a_{4}^{*}\left(\theta \right)$, $a_{\gamma }\left(\theta \right)$ is the steering vector for θ, and γ=1,2,3,4.

**Property** **3.** *Define $H=\left\{|D|:D=f\left(S\right),S=\left\{n_{1},\ldots ,n_{M}\right\},\forall n_{1},\ldots ,n_{M}\in R^{3}\right\}$, where f(·) is the function that S maps to D by Definition 1. Then the supremum of H is*

(7) $$\begin{array}{c} \sup\left\{H\right\}=\left(M^{4}-2M^{3}+7M^{2}-6M+4\right)/4. \end{array}$$

Properties 1, 2, and 3 are proved in Appendix A, Appendix B, and Appendix C, respectively.

From Property 2, we have $B={\mathrm{JA}}_{D}$, where $A_{D}=\left[a_{D}\left(\theta_{1}\right),a_{D}\left(\theta_{2}\right),\ldots ,a_{D}\left(\theta_{K}\right)\right]$. Substituting it to (3), we obtain $c_{x}={\mathrm{JA}}_{D}c_{s}$. Due to Property 1, we have

(8) $$\begin{array}{c} J^{+}c_{x}=A_{D}c_{s}\in C^{\left|D\right|}, \end{array}$$

where $J^{+}={\left(J^{H}J\right)}^{-1}J^{H}$ is the Moore–Penrose inverse of J. Comparing the model (8) with (3), it is clear that the row dimension decreases from $M^{4}$ to |D|.

*Remark 1:* An analogous procedure can be found in covariance vector-based DOA estimation methods [22]. Among HOCs-based DOA estimation methods, rectangular 2q-MUSIC provides two strategies to reduce the dimension of a rectangle FOCs matrix (i.e., selection strategy and averaging strategy), by eliminating redundancies of associated left and right virtual steering vectors [7]. From this perspective, the proposed dimension reduction procedure is the essential realization of the averaging strategy applying to FODCA, with an explicit formulation (8). It is clear that the averaging strategy is better than the selection strategy due to the averaging of the noise in the associated observation components [7], if the computation burden is not taken into consideration. Thus, the averaging strategy can also be treated as a denoising procedure. In addition, it can be seen that FODCA has the same geometry as a fourth-order virtual array with parameters (q,l)=(4,2) [25], which is deduced from an eighth-order cumulants-based array processing problem. In TABLE IX from [25], the max number of identical virtual sensors in the fourth-order virtual array, with parameters (q,l)=(4,2) and space diversity (without angular and polarization diversity), is illustrated, and it is equal to (7) after simplification due to the same geometry. The detailed derivation of (7) is provided in Appendix C, since it is not provided in [25].

*Remark 2:* It is clear that when 2≤K<M, the limiting root mean square error (RMSE) of covariance vector-based methods is not necessarily zero [22,26], while the RMSE of the traditional MUSIC method converges to zero as signal–noise-ratio (SNR) approaches infinity. As the FODCA can be regarded as two levels of second-order difference co-array [8,18], applied to construct covariance vector-based methods, it can be deduced that the FOCs vector-based DOA estimation methods, including [8,16,17,18], will be outperformed by traditional MUSIC in high-SNR regions when 2≤K<M. From this point, we only suggest to use these methods (including the proposed method) in underdetermined cases (K≥M).

### 3.2. Sparse Model

In this subsection we develop the sparse model from (8). Let $\tilde{\theta }=\left[{\tilde{\theta }}_{1},\ldots ,{\tilde{\theta }}_{I}\right]$ be a fixed sampling grid in the DOA range, where I≫|D|. Let $\tilde{\theta }$ be a uniform grid with a grid resolution $r={\tilde{\theta }}_{2}-{\tilde{\theta }}_{1}\propto I^{-1}$. Suppose ${\tilde{\theta }}_{w_{k}}$ is the nearest grid point to $\theta_{k}$, where $w_{k}\in \left\{1,\ldots ,I\right\}$. Then, the steering vector $a_{D}\left(\theta_{k}\right)$ can be approximated by its first-order Taylor series

(9) $$\begin{array}{c} a_{D}\left(\theta_{k}\right)\approx a_{D}\left({\tilde{\theta }}_{w_{k}}\right)+a_{D}^{\prime }\left({\tilde{\theta }}_{w_{k}}\right)\left(\theta_{k}-{\tilde{\theta }}_{w_{k}}\right). \end{array}$$

Denote

(10) $$\begin{array}{c} {\tilde{A}}_{D}=\left[a_{D}\left({\tilde{\theta }}_{1}\right),\ldots ,a_{D}\left({\tilde{\theta }}_{I}\right)\right], \end{array}$$

(11) $$\begin{array}{c} {\tilde{A}}_{D}^{\prime }=\left[a_{D}^{\prime }\left({\tilde{\theta }}_{1}\right),\ldots ,a_{D}^{\prime }\left({\tilde{\theta }}_{I}\right)\right]. \end{array}$$

Denote $\beta ={\left[\beta_{1},\ldots ,\beta_{I}\right]}^{T}\in {\left[-r/2,r/2\right]}^{I}$ and $\Phi ={\tilde{A}}_{D}+{\tilde{A}}_{D}^{\prime }\mathrm{diag}\left\{\beta \right\}$, where for w=1,…,I, $\beta_{w}=\theta_{k}-{\tilde{\theta }}_{w_{k}}$ if $w=w_{k}$ for any k=1,…,K and $\beta_{w}=0$ otherwise, and diag{·} denotes the operator to form a diagonal matrix by a vector or to form a vector by diagonal entries of a matrix. Neglecting approximation errors of (9), model (8) can be expressed as:

(12) $$\begin{array}{c} J^{+}c_{x}=\Phi z, \end{array}$$

where $z={\left[z_{1},z_{2},\ldots ,z_{I}\right]}^{T}$ is the zero-padded extension of $c_{s}$ from $\left[\theta_{1},\ldots ,\theta_{K}\right]$ to $\left[{\tilde{\theta }}_{1},\ldots ,{\tilde{\theta }}_{I}\right]$.

Consider the case of existing estimation errors of FOCs. Let ${\hat{c}}_{x}=c_{x}+e$, where ${\hat{c}}_{x}$ is the estimate of $c_{x}$, and e is the estimation errors vector. Then, model (12) becomes

(13) $$\begin{array}{c} J^{+}{\hat{c}}_{x}=\Phi z+J^{+}e. \end{array}$$

Assume $e∼\mathrm{AsCN}(0_{M^{4}},Q)$ [1], where AsCN denotes the asymptotic normal distribution, $0_{M^{4}}$ denotes zero vector with $M^{4}$ elements, and $Q\in C^{M^{4}\times M^{4}}$ is a positive definite (Hermitian) matrix. The estimation of FOCs and the matrix Q are discussed in Section 3.3. Following this assumption, we have $J^{+}e∼\mathrm{AsCN}\left(0_{\left|D\right|},\tilde{Q}\right)$, where $\tilde{Q}=J^{+}Q{\left(J^{+}\right)}^{T}$. Furthermore, we can obtain ${\tilde{Q}}^{-\frac{1}{2}}J^{+}e∼\mathrm{AsCN}\left(0_{\left|D\right|},I_{\left|D|\times |D\right|}\right)$, where $I_{\left|D|\times |D\right|}$ is |D| dimensional identity matrix. We denote by

(14) $$\begin{array}{c} {\tilde{c}}_{x}={\tilde{Q}}^{-\frac{1}{2}}J^{+}{\hat{c}}_{x}\in C^{\left|D\right|}, \end{array}$$

(15) $$\begin{array}{cc} \tilde{\Phi }={\tilde{Q}}^{-\frac{1}{2}}\Phi & ={\tilde{Q}}^{-\frac{1}{2}}\left({\tilde{A}}_{D}+{\tilde{A}}_{D}^{\prime }\mathrm{diag}\left\{\beta \right\}\right)\in C^{|D|\times I}, \end{array}$$

(16) $$\begin{array}{c} \tilde{e}={\tilde{Q}}^{-\frac{1}{2}}J^{+}e\in C^{\left|D\right|}. \end{array}$$

Then, we can obtain the following SMV off-grid sparse model

(17) $$\begin{array}{c} {\tilde{c}}_{x}=\tilde{\Phi }z+\tilde{e}, \end{array}$$

with $\tilde{e}∼\mathrm{AsCN}\left(0_{\left|D\right|},I_{\left|D|\times |D\right|}\right)$. Note that if setting $\beta_{w}=0$ for w=1,…,I, the model (17) is simplified to an on-grid DOA model.

### 3.3. Estimation of FOCs and the Matrix Q

To facilitate the implementation of the proposed method, in this subsection we present the estimation methods of FOCs and the matrix Q.

Let us introduce the following general notations: $m_{4,x}\left(i_{1},i_{2},i_{3}*,i_{4}*\right)=E\left\{x_{i_{1}}\left(t\right)x_{i_{2}}\left(t\right)x_{i_{3}}^{*}\left(t\right)x_{i_{4}}^{*}\left(t\right)\right\}$, $m_{2,x}\left(i_{1},i_{2}*\right)=E\left\{x_{i_{1}}\left(t\right)x_{i_{2}}^{*}\left(t\right)\right\}$, and $m_{2,x}\left(i_{1},i_{2}\right)=E\left\{x_{i_{1}}\left(t\right)x_{i_{2}}\left(t\right)\right\}$. Their empirical estimators are ${\hat{m}}_{4,x}\left(i_{1},i_{2},i_{3}*,i_{4}*\right)=\frac{1}{L}\sum_{t=1}^{L}\left\{x_{i_{1}}\left(t\right)x_{i_{2}}\left(t\right)x_{i_{3}}^{*}\left(t\right)x_{i_{4}}^{*}\left(t\right)\right\}$, ${\hat{m}}_{2,x}\left(i_{1},i_{2}*\right)=\frac{1}{L}\sum_{t=1}^{L}\left\{x_{i_{1}}\left(t\right)x_{i_{2}}^{*}\left(t\right)\right\}$, and ${\hat{m}}_{2,x}\left(i_{1},i_{2}\right)=\frac{1}{L}\sum_{t=1}^{L}\left\{x_{i_{1}}\left(t\right)x_{i_{2}}\left(t\right)\right\}$, respectively, where *L* is the number of snapshots. Due to the assumptions of sources and noises, ${\left[c_{x}\right]}_{i}$ can be expressed as [25]:

(18) $$\begin{array}{c} {\left[c_{x}\right]}_{i}=\mathrm{cum}\left\{x_{i_{1}}\left(t\right),x_{i_{2}}\left(t\right),x_{i_{3}}^{*}\left(t\right),x_{i_{4}}^{*}\left(t\right)\right\}\\ =m_{4,x}\left(i_{1},i_{2},i_{3}*,i_{4}*\right)-m_{2,x}\left(i_{1},i_{2}\right)m_{2,x}^{*}\left(i_{3},i_{4}\right)\\ -m_{2,x}\left(i_{1},i_{3}*\right)m_{2,x}\left(i_{2},i_{4}*\right)-m_{2,x}\left(i_{1},i_{4}*\right)m_{2,x}\left(i_{2},i_{3}*\right). \end{array}$$

Utilizing the corresponding empirical estimators of terms in (18), ${\left[c_{x}\right]}_{i}$ can be estimated by

(19) $$\begin{array}{c} {\left[{\hat{c}}_{x}\right]}_{i}={\hat{m}}_{4,x}\left(i_{1},i_{2},i_{3}*,i_{4}*\right)-{\hat{m}}_{2,x}\left(i_{1},i_{2}\right)m_{2,x}^{*}\left(i_{3},i_{4}\right)\\ -{\hat{m}}_{2,x}\left(i_{1},i_{3}*\right)m_{2,x}\left(i_{2},i_{4}*\right)-{\hat{m}}_{2,x}\left(i_{1},i_{4}*\right)m_{2,x}\left(i_{2},i_{3}*\right). \end{array}$$

Consider complex variables $X\left(t\right)=x_{i_{1}}\left(t\right)x_{i_{2}}\left(t\right)x_{i_{3}}^{*}\left(t\right)x_{i_{4}}^{*}\left(t\right)-x_{i_{1}}\left(t\right)x_{i_{2}}\left(t\right)\sum_{t_{0}=1}^{L}x_{i_{3}}^{*}\left(t_{0}\right)x_{i_{4}}^{*}\left(t_{0}\right)/L-x_{i_{1}}\left(t\right)x_{i_{3}}^{*}\left(t\right)\sum_{t_{1}=1}^{L}x_{i_{2}}\left(t_{1}\right)x_{i_{4}}^{*}\left(t_{1}\right)/L-x_{i_{1}}\left(t\right)x_{i_{4}}^{*}\left(t\right)\sum_{t_{2}=1}^{L}x_{i_{2}}\left(t_{2}\right)x_{i_{3}}^{*}\left(t_{2}\right)/L$ for t=1,2,…,L. It is obvious that $\lim_{L\to +\infty }E\left\{X\left(t\right)\right\}={\left[c_{x}\right]}_{i}$. According to central limit theorem, as L→+∞, the sample average $\bar{X}=\sum_{t=1}^{L}X\left(t\right)={\left[{\hat{c}}_{x}\right]}_{i}$ converges in distribution to a complex normal. This solution theoretically supports the assumption that $e={\hat{c}}_{x}-c_{x}∼\mathrm{AsCN}\left(0_{M^{4}},Q\right)$ in Section 3.2, and we aim for a robust estimation of Q in the following derivation.

Consider signals given by

(20) $$\begin{array}{c} y(t)=x(t)⊗x(t)-E\{x(t)⊗x(t)\}. \end{array}$$

It is obvious that $E\left\{y\left(t\right)\right\}=0_{M^{2}}$. Let $y_{g}\left(t\right)$ denote the *g*th element of y(t), $g_{1}=\left(i_{1}-1\right)M+i_{2}$, and $g_{2}=\left(i_{3}-1\right)M+i_{4}$, then we have

(21) $$\begin{array}{ccc} m_{2,y}\left(g_{1},g_{2}*\right)= & m_{4,x}\left(i_{1},i_{2},i_{3}*,i_{4}*\right)- & m_{2,x}\left(i_{1},i_{2}\right)m_{2,x}^{*}\left(i_{3},i_{4}\right). \end{array}$$

Rewrite (18) as:

(22) $$\begin{array}{c} {\left[c_{x}\right]}_{i}=m_{2,y}\left(g_{1},g_{2}*\right)-m_{2,x}\left(i_{1},i_{3}*\right)m_{2,x}\left(i_{2},i_{4}*\right)\\ -m_{2,x}\left(i_{1},i_{4}*\right)m_{2,x}\left(i_{2},i_{3}*\right), \end{array}$$

where y(t) is given by (20).

**Lemma** **1.** *Given two joint distributed complex-valued random variables $Y_{t}$ and $Z_{t}$ with snapshots index t=1,2,…,L, $Y_{t}$, and $Z_{t}$ are independent from snapshot to snapshot, respectively. As L approaches infinity, it holds true that*

(23) $$\begin{array}{c} \mathrm{var}\left(\bar{Y_{t}Z_{t}}\right)/\mathrm{var}\left({\bar{Y}}_{t}{\bar{Z}}_{t}\right)=O\left(L\right), \end{array}$$

*where var(·) is variance, and $\bar{Y_{t}Z_{t}}$, ${\bar{Y}}_{t}$, and ${\bar{Z}}_{t}$ are sample averages of $Y_{t}Z_{t}$, $Y_{t}$, and $Z_{t}$ with L snapshots, respectively.*

**Proof.** See Appendix D.

Due to Lemma 1, from (19) and (22), it is easy to find that when the snapshots number *L* is large, the term ${\hat{m}}_{2,y}\left(g_{1},g_{2}*\right)$ has much larger variance than the other two terms in (22). This implies that the estimation error of ${\left[{\hat{c}}_{x}\right]}_{i}$ is mainly caused by the term ${\hat{m}}_{2,y}\left(g_{1},g_{2}*\right)$, giving rise to the following approximate expression of $e_{i}$:

(24) $$\begin{array}{c} e_{i}={\left[{\hat{c}}_{x}\right]}_{i}-{\left[c_{x}\right]}_{i}\approx {\hat{m}}_{2,y}\left(g_{1},g_{2}*\right)-m_{2,y}\left(g_{1},g_{2}*\right). \end{array}$$

Since the signals are independent from snapshot to snapshot, and $E\left\{y\left(t\right)\right\}=0_{M^{2}}$, we can obtain

(25) $$\begin{array}{cc} E\{e_{i}e_{j}^{*}\} & \approx \frac{1}{L^{2}}\sum_{t_{1}=1}^{L}\sum_{t_{2}=1}^{L}E\left\{\left[y_{g_{1}}\left(t_{1}\right)y_{g_{2}}^{*}\left(t_{1}\right)\right]{\left[y_{h_{1}}\left(t_{2}\right)y_{h_{2}}^{*}\left(t_{2}\right)\right]}^{*}\right\}\\ & -m_{2,y}\left(g_{1},g_{2}*\right)m_{2,y}^{*}\left(h_{1},h_{2}*\right)\\ & =\mathrm{cum}\{y_{g_{1}}\left(t\right),y_{g_{2}}^{*}\left(t\right),y_{h_{1}}^{*}\left(t\right),y_{h_{2}}\left(t\right)\}/L\\ & +m_{2,y}\left(g_{1},h_{1}*\right)m_{2,y}\left(g_{2}*,h_{2}\right)/L\\ & +m_{2,y}\left(g_{1},h_{2}\right)m_{2,y}\left(g_{2}*,h_{1}*\right)/L, \end{array}$$

where $h_{1}=\left(j_{1}-1\right)M+j_{2}$, $h_{2}=\left(j_{3}-1\right)M+j_{4}$, $j=\left(h_{1}-1\right)M^{2}+h_{2}$, and $j_{1},j_{2},j_{3},j_{4}=1,2,\ldots ,M$.

In (25), the term $\mathrm{cum}\{y_{g_{1}}\left(t\right),y_{g_{2}}^{*}\left(t\right),y_{h_{1}}^{*}\left(t\right),y_{h_{1}}^{*}\left(t\right)\}/L$ contains the eighth-order statistics of x(t), which significantly aggravates the inaccuracy of estimation if the number of snapshots is not large enough. In addition, as the number of snapshots number approaches infinity, we get $E\{e_{i}e_{j}^{*}\}\approx 0$. Therefore, we neglect this term to obtain a robust estimation of Q, given by

(26) $$\begin{array}{c} {\hat{Q}}_{i,j}={\hat{m}}_{2,y}\left(g_{1},h_{1}*\right){\hat{m}}_{2,y}\left(g_{2}*,h_{2}\right)/L\\ +{\hat{m}}_{2,y}\left(g_{1},h_{2}\right){\hat{m}}_{2,y}\left(g_{2}*,h_{1}*\right)/L. \end{array}$$

Rewrite (26) in matrix form

(27) $$\begin{array}{c} \hat{Q}={\hat{R}}_{y}⊗{\hat{R}}_{y}^{T}/L+\left[{\hat{T}}_{y}⊗{\hat{T}}_{y,1}^{*},{\hat{T}}_{y}⊗{\hat{T}}_{y,2}^{*},\ldots ,{\hat{T}}_{y}⊗{\hat{T}}_{y,M^{2}}^{*}\right]/L, \end{array}$$

where ${\hat{R}}_{y}=\sum_{t=1}^{L}y\left(t\right)y^{H}\left(t\right)/L$, ${\hat{T}}_{y}=\sum_{t=1}^{L}y\left(t\right)y^{T}\left(t\right)/L$, and ${\hat{T}}_{y,h_{1}}$ is the $h_{1}$th column of ${\hat{T}}_{y}$.

Note that the estimator $\hat{Q}$ in (30) need to be revised in certain cases. It is clear that, for the noise-free case, ${\hat{R}}_{y}=\left(A⊗A\right)\sum_{t=1}^{L}\left\{\left[s\left(t\right)s^{H}\left(t\right)\right]⊗\left[s\left(t\right)s^{H}\left(t\right)\right]\right\}{\left(A⊗A\right)}^{H}/L$, and ${\hat{T}}_{y}=\left(A⊗A\right)\sum_{t=1}^{L}\left\{\left[s\left(t\right)s^{T}\left(t\right)\right]⊗\left[s\left(t\right)s^{T}\left(t\right)\right]\right\}{\left(A⊗A\right)}^{T}/L$. When K<M, the ranks of both matrices ${\hat{R}}_{y}$ and ${\hat{T}}_{y}$ are $K^{2}$. As a result, the estimator (27) may be singular, which may lead to a singular matrix $\tilde{Q}$. This is unallowable for model (17). Therefore, when K<M, and SNR is high, to avoid above problems, Q is estimated by

(28) $$\begin{array}{c} \hat{Q}={\hat{R}}_{y}⊗{\hat{R}}_{y}^{T}/L+\left[{\hat{T}}_{y}⊗{\hat{T}}_{y,1}^{*},{\hat{T}}_{y}⊗{\hat{T}}_{y,2}^{*},\ldots ,{\hat{T}}_{y}⊗{\hat{T}}_{y,M^{2}}^{*}\right]/L+\epsilon_{1}I_{\left|D|\times |D\right|}, \end{array}$$

where $\epsilon_{1}$ is a small positive number.

Specially, if the observed signals are circular, we have $m_{2,x}\left(l_{1},l_{2}\right)=0$ and $m_{4,x}\left(l_{1},l_{2},l_{3},l_{4}\right)=0$ for $\forall l_{1},l_{2},l_{3},l_{4}=1,\ldots ,M$. Then, we obtain y(t)=x(t)⊗x(t), and $c_{i}$ can be estimated by

(29) $$\begin{array}{c} {\left[{\hat{c}}_{x}\right]}_{i}={\hat{m}}_{4,x}\left(i_{1},i_{2},i_{3}*,i_{4}*\right)-{\hat{m}}_{2,x}\left(i_{1},i_{3}*\right){\hat{m}}_{2,x}\left(i_{2},i_{4}*\right)\\ -{\hat{m}}_{2,x}\left(i_{1},i_{4}*\right){\hat{m}}_{2,x}\left(i_{2},i_{3}*\right). \end{array}$$

Furthermore, it is obvious that $m_{2,y}\left(g_{1},h_{2}\right)$ and $m_{2,y}\left(g_{2}*,h_{1}*\right)$ become zero in (25). Then, the estimator of Q in (27) becomes:

(30) $$\begin{array}{c} \hat{Q}={\hat{R}}_{y}⊗{\hat{R}}_{y}^{T}/L. \end{array}$$

Accordingly, when K<M and SNR is high, for circular sources, Q is estimated by

(31) $$\begin{array}{c} \hat{Q}={\hat{R}}_{y}⊗{\hat{R}}_{y}^{T}/L+\epsilon_{2}I_{\left|D|\times |D\right|}, \end{array}$$

where $\epsilon_{2}$ is a small positive number.

*Remark 3:* Approximate expressions for the covariances of FOCs which are more precise than (25) are derived in [5]. However, it is not easy to use in our method in practice, because several higher-order (greater than four) moments need to be estimated, which may cause the estimation of Q to be not as robust as FOCs. For example, if the number of snapshots is large enough to generate precise estimates of FOCs, but not large enough to generate ones of higher-order (greater than four) moments, the DOA estimation results may suffer from a mass of outliers in independent experiments. In comparison, (26) contains at most fourth-order statistics, which means that it is more robust and has lower computational cost. We will show that our estimators work well for the proposed method in numerical simulations.

### 3.4. DOA Estimation by OGSBI Method

In this subsection, the sparse model (17) is solved to obtain the DOAs by the off-grid sparse Bayesian inference (OGSBI) method. Recall the sparse model (17):

(32) $$\begin{array}{c} {\tilde{c}}_{x}=\tilde{\Phi }z+\tilde{e}. \end{array}$$

In Section 3.3, we showed that the estimation errors of FOCs obey $e={\hat{c}}_{x}-c_{x}∼\mathrm{AsCN}\left(0_{M^{4}},Q\right)$, and the robust estimators of Q are also given. As $\tilde{e}={\tilde{Q}}^{-\frac{1}{2}}J^{+}e\in C^{\left|D\right|}$, it is obvious that $\tilde{e}∼\mathrm{AsCN}\left(0_{\left|D\right|},I_{\left|D|\times |D\right|}\right)$. Following the sparse Bayesian formulation in [21], further assume $\tilde{e}∼\mathrm{CN}\left(0_{\left|D\right|},I_{\left|D|\times |D\right|}\right)$ for simplicity. Then, we have

(33) $$\begin{array}{c} p\left({\tilde{c}}_{x}|z,\beta \right)=\mathrm{CN}\left(\tilde{\Phi }z,I_{\left|D|\times |D\right|}\right). \end{array}$$

Adopt the two-stage hierarchical prior for z that p(z;ρ)=∫p(z|α)p(α;ρ)dα, in which

(34) $$\begin{array}{c} p\left(z|\alpha \right)=\mathrm{CN}\left(0_{I},\Lambda \right), \end{array}$$

(35) $$\begin{array}{c} p\left(\alpha ;\rho \right)=\prod_{w=1}^{I}\Gamma \left(\alpha_{w};1,\rho \right), \end{array}$$

where ρ>0, $\alpha \in R^{I}$, Λ=diag{α}, $\Gamma \left(\alpha_{w};1,\rho \right)=\rho e^{-\rho \alpha_{w}}$ is the probability density function (PDF) of the Gamma distribution with parameters (1,ρ), and $\alpha_{w}$ is the *w*th entry of α. Here the Gamma hyperprior is assumed for $\alpha_{w}$, since it is a conjugate prior of the Gaussian distribution, and it is widely used in SBL techniques and demonstrated with good performance and robustness [13,21,27,28].

Assume a uniform prior for β: $\beta ∼U({\left[-r/2,r/2\right]}^{I})$. By combining the stages of the hierarchical Bayesian model, the joint PDF is

(36) $$\begin{array}{c} p\left(z,{\tilde{c}}_{x},\alpha ,\beta \right)=p\left({\tilde{c}}_{x}|z,\beta \right)p\left(z|\alpha \right)p\left(\alpha \right)p\left(\beta \right). \end{array}$$

The posterior distribution of z can be obtained

(37) $$\begin{array}{c} p\left(z|{\tilde{c}}_{x},\Lambda ,\beta \right)=\mathrm{CN}\left(\mu ,\Sigma \right), \end{array}$$

with

(38) $$\begin{array}{c} \mu =\Sigma {\tilde{\Phi }}^{H}{\tilde{c}}_{x} \end{array}$$

(39) $$\begin{array}{cc} \Sigma ={\left({\tilde{\Phi }}^{H}\tilde{\Phi }+\Lambda^{-1}\right)}^{-1} & =\Lambda -{\Lambda \tilde{\Phi }}^{H}{\left(I+{\tilde{\Phi }\Lambda \tilde{\Phi }}^{H}\right)}^{-1}\tilde{\Phi }\Lambda . \end{array}$$

As a single snapshot case of the OGSBI method, we can obtain the following updates of α from the deduction in [21]:

(40) $$\begin{array}{c} \alpha_{w}^{\mathrm{new}}=\left(\sqrt{1+4\rho E\{|z_{w}{|}^{2}\}}-1\right)/\left(2\rho \right), \end{array}$$

where $E\{|z_{w}{|}^{2}\}=|\mu_{w}{|}^{2}+\Sigma_{ww}$, $\mu_{w}$ is the *w*th entry of μ, and $\Sigma_{ww}$ is (w,w)th entry of Σ. For β, by maximizing $E\{\log p\left({\tilde{c}}_{x}|z,\beta \right)p\left(\beta \right)\}$, we can obtain the updates

(41) $$\begin{array}{c} \beta^{\mathrm{new}}=\underset{\beta \in {\left[-r/2,r/2\right]}^{I}}{\arg\min}\left\{\beta^{T}P\beta -2q^{T}\beta \right\}, \end{array}$$

where $P=R\{{\left[{\left({\tilde{A}}_{D}^{\prime }\right)}^{H}{\tilde{A}}_{D}^{\prime }\right]}^{*}⊙\left({\mu \mu }^{H}+\Sigma \right)\}$, $q=R\{\mathrm{diag}\left\{\mu^{*}\right\}{\left({\tilde{A}}_{D}^{\prime }\right)}^{H}\left({\tilde{c}}_{x}-{\tilde{A}}_{D}\mu \right)\}-R\{\mathrm{diag}\{{\left({\tilde{A}}_{D}^{\prime }\right)}^{H}{\tilde{A}}_{D}\Sigma \}\}$, R(·) takes the real part of a complex variable and ⊙ is the Hadamard product. An easier way to estimate β is provided in [21]. We use β, P, and q hereafter to denote their truncated versions for simplicity. By (41) and $\frac{\partial }{\partial \beta }\left\{\beta^{T}P\beta -2q^{T}\beta \right\}=2\left(P\beta -q\right)$, we have $\beta^{\mathrm{new}}=\overset{ˇ}{\beta }$ if P is invertible and $\overset{ˇ}{\beta }=P^{-1}q\in {\left[-r/2,r/2\right]}^{K}$. Otherwise, we update β elementwise. That is, at each step we update one $\beta_{w}$ by fixing up the other entries of β. For w=1,…,K, we first let $\overset{ˇ}{\beta }=\frac{q_{k}-{\left(P_{k}\right)}_{-k}^{T}\beta_{-k}}{P_{kk}}$, where $\beta_{-k}$ denotes β without the *k*th entry. Then, by constraining $\beta_{k}\in \left[-r/2,r/2\right]$, we have

(42) $$\beta_{k}^{\mathrm{new}}=\{\begin{array}{c} {\overset{ˇ}{\beta }}_{k},\;\;\;\;\;\;\mathrm{if}\;{\overset{ˇ}{\beta }}_{k}\in \left[-r/2,r/2\right];\\ -r/2,\;\mathrm{if}\;{\overset{ˇ}{\beta }}_{k}<-r/2;\\ r/2,\;\;\;\;\mathrm{otherwise}. \end{array}$$

The OGSBI is terminated if $∥\alpha^{\kappa +1}-\alpha^{\kappa }{∥}_{2}/{∥\alpha^{\kappa }∥}_{2}<\tau$ or the maximum number of iterations is reached, where κ is the iteration and τ is the predefined tolerance parameter. Then, α can be treated as the pseudo-spectrum

(43) $$\begin{array}{c} P_{w}=\alpha_{w}. \end{array}$$

By finding the grid indices of highest *K* peaks of P, denoted by ${\hat{p}}_{k},k=1,\ldots ,K$, we can obtain *K* estimated DOAs by

(44) $$\begin{array}{c} {\hat{\theta }}_{k}={\hat{\theta }}_{w_{k}}+{\hat{\beta }}_{w_{k}}. \end{array}$$

Note that the parameter ρ in the method should be given in advance. According to associated information in [21] and our testing results, the OGSBI method is insensitive to ρ if ρ is not too large. Furthermore, we have observed that by setting ρ=0.01, the sparse Bayesian learning methods in [13,21,28] always achieve a good performance in a wide range of scenarios. By contrast, the 4-L1 method [16,17,18] is sensitive to the parameter of allowable error bound, which is not easy to estimate (it was chosen to give the best results through trial-and-error for each scenario in the corresponding simulations in [16,17,18]). Therefore, compared with the 4-L1 method, the OGSBI method has the advantage of convenience in setting parameters.

Finally, we summarize our method in Algorithm 1.

**Algorithm 1**   The proposed sparse method using denoised FOCs vector1)Input: x, S, and *d*.2)Initialization: *r*, α, ρ, ϵ, and τ.3)According to the associated scenarios, calculate ${\hat{c}}_{x}$ by (19) or (29).4)According to the associated scenarios, calculate $\hat{Q}$ by (27), (28), (30), or (31).5)Calculate J, ${\tilde{A}}_{D}$, ${\tilde{A}}_{D}^{\prime }$, and ${\tilde{c}}_{x}$ by Definition 2, (10), (11), and (14), respectively.6)Repeat the following:
 a)  Calculate μ and Σ by (38) and (39), respectively.
 b)  Update α by (40).
 c)  Update β by (42).
 d)  If $∥\alpha^{\kappa +1}-\alpha^{\kappa }{∥}_{2}/{∥\alpha^{\kappa }∥}_{2}<\tau$ or the maximum number of iterations is reached, iteration terminates.7)Find the grid indices of the highest peaks of (43), and output the DOAs by (44).

### 3.5. Identifiability of the Number of Sources

According to the theorems about parameter identifiability in [29,30,31] and the model (8), we have the following proposition.

**Proposition** **1.** *Any K sources can be uniquely identified from model (17) if and only if*

(45) $$\begin{array}{c} K<\mathrm{spark}\{B_{\theta }\}/2, \end{array}$$

*where $\mathrm{spark}\{B_{\theta }\}$ is defined as the smallest number of elements in $B_{\theta }$ that are linearly dependent [32], $B_{\theta }=\left\{b\left(\theta \right):\theta \in \Theta \right\}\subset C^{\left|D\right|}$, and* Θ *is [−π/2,π/2) for linear array and [−π,π) for planar array.*

It is generally difficult to compute $\mathrm{spark}\{B_{\theta }\}$, except for when D is a uniform linear array (ULA). In this circumstance, D is hole-free, $\mathrm{spark}\{B_{\theta }\}=|D|+1$, and then K<(|D|+1)/2 sources can be identified. However, hole-free FODCA with $O(M^{4})$ DOFs has not been studied so far to the best of our knowledge. Recently, [16,17,18] provided several methods to construct FODCA with larger ULA segments, with which $K<(|D_{\mathrm{ULA}}|+1)/2$ sources can be identified, where $D_{\mathrm{ULA}}$ is defined as the largest ULA segment in D.

For general arrays, because $B_{\theta }\subset C^{\left|D\right|}$, it is obvious that

(46) $$\begin{array}{c} \mathrm{spark}\{B_{\theta }\}\leq |D|+1. \end{array}$$

According to Property 3, (46), and Proposition 1, we can obtain a necessary condition regarding the identifiability of the number of sources.

**Theorem** **1.** *Consider model (17), a necessary condition to uniquely identify all sources is that the number of sources K fulfills*

(47) $$\begin{array}{c} K\leq (M^{4}-2M^{3}+7M^{2}-6M)/8. \end{array}$$

**Proof.** See Appendix E. 

In other words, any more than $(M^{4}-2M^{3}+7M^{2}-6M)/8$ sources cannot be uniquely identified by model (17).

In addition, there are $O(M^{4})$ distinct elements in $D_{\mathrm{ULA}}$ for certain sparse linear arrays (SLAs), such as four-level nested arrays [8] and arrays constructed from a expanding and shift scheme which consists of coprime arrays or nested arrays [18]. Therefore, our proposed method can identify $O(M^{4})$ sources at most, if these arrays (not limited to) are used.

### 3.6. Complexity

Consider the worst case of complexity. For non-circular sources, the estimation of $c_{x}$ using (19) needs $O(LM^{4})$ multiplications. The estimation of Q using (27) or (28) needs $O(LM^{8})$ multiplications. Note that all the symmetries of $c_{x}$ and Q are not taken into account here, while it does not affect the order of complexity. Each iteration of (38) and (39) needs $O(I^{2}|D|)$ multiplications, which are much more than each iteration of (40) and (42). Let *T* denote the iteration number, and consider the worst case that $O\left(|D|\right)=O\left(M^{4}\right)$. Combing all of the above processes, we can obtain that the complexity of the proposed method is $O(LM^{8}+I^{2}M^{4}T)$.

### 3.7. Comparison with State-of-the-Art Methods

In this subsection, we compare the proposed method with state-of-the-art FOCs-based ones, in terms of required geometry, identifiability, prior knowledge of the number of sources, ability to handle correlated sources, numerical complexity, and sensitivity to parameters. Associated methods are listed as follows:

- 4-MUSIC [5,6]: non-redundant version with averaging strategy [7], denoted by A-4-MUSIC;
- 4-SS-MUSIC [8]: with averaging strategy, denoted by A-4-SS-MUSIC;
- 4-L1 [16,17,18].

Associated comparison results are illustrated in Table 1.

In Table 1, $T_{1}$ and $T_{2}$ denote the iteration number of the proposed method and 4-L1, respectively. All comparison results are obvious or were discussed in the previous subsections, except for the comparison of complexity. Recall that *M* is the number of sensors, *L* is the number of snapshots, and *I* is the number of grid points. It should be noted that the complexities of four methods are derived under the assumption that $O\left(|D|\right)=O\left(M^{4}\right)$, which gives the best identifiability and highest complexities for all methods. Due to the condition I≫|D| required to perform the sparsity in the proposed method and 4-L1, it is clear that $O\left(I\right)\geq O\left(M^{4}\right)$, which results in $O\left(I^{3}T_{2}\right)>O\left(IM^{8}\right)$ and $O\left(I^{2}M^{4}T_{1}\right)>O\left(IM^{8}\right)$. Consequently, the proposed method and 4-L1 always have higher complexity than A-4-MUSIC and A-4-SS-MUSIC.

Next, we make a comparison of complexity between the proposed method and 4-L1. Firstly, if the number of snapshots *L* is large enough that the terms $O(I^{3}T_{2})$ and $O(I^{2}M^{4}T_{1})$ can be negligible, the proposed method has higher complexity than 4-L1 due to the term $O(LM^{8})$ which is caused by the estimation of Q. Secondly, if *L* is small, we limit the comparison between $O(I^{3}T_{2})$ and $O(I^{2}M^{4}T_{1})$. It is clear that $O\left(I^{3}\right)\geq O\left(I^{2}M^{4}\right)$, implying that the proposed method has lower complexity than 4-L1 in one iteration. However, it is a fact that 4-L1 always has less iterations than the proposed method (i.e., $T_{1}>T_{2}$). Thus, it is not easy to draw a conclusion. In fact, to achieve the super resolution, 4-L1 needs a dense grid, while the proposed method only needs a coarser grid due to the off-grid parameter estimation, which gives our method an advantage for complexity comparison. It should be noted that off-grid parameter estimation technique has been successfully applied to L1-norm minimization methods in [33,34]. However, to the best of our knowledge, the off-grid version of the 4-L1 method has not been published, and it is beyond the scope of this paper.

## 4. Numerical Simulations

Numerical simulations were carried out to demonstrate the superior performance of the proposed method. In the following simulations, the sampled analytic signals of statistically independent quaternary phase-shift keying (QPSK) signals were used as circular sources. The RMSE was chosen as the performance criterion, and is computed by

(48) $$\begin{array}{c} RMSE=\sqrt{\frac{1}{FK}\sum_{f=1}^{F}\sum_{k=1}^{K}{\left|{\hat{\theta }}_{k,f}-\theta_{k}\right|}^{2}}, \end{array}$$

where *F* is the number of independent experiments, and ${\hat{\theta }}_{k,f}$ is the estimate of $\theta_{k}$ in the *f*th independent experiment. The RMSE performances of the four methods in Table 1 with respect to number of snapshots, grid, adjacent sources, SNR, modelling errors, geometry, and circularity of sources were further investigated. In the proposed method, we set that ρ=0.01 and $\tau =10^{-4}$ for all scenarios, and set $\epsilon_{1}=10^{-5}$ or $\epsilon_{2}=10^{-5}$ for the case that K<M and SNR≥8 dB. 4-L1 was solved by the SeDuMi toolbox [35], and the associated allowable error bound was chosen to give the best results through trial-and-error for each scenario as done in [16,17,18]. Each simulation executed F=200 independent experiments for all methods.

### 4.1. Identifiability

**Figure 1:**
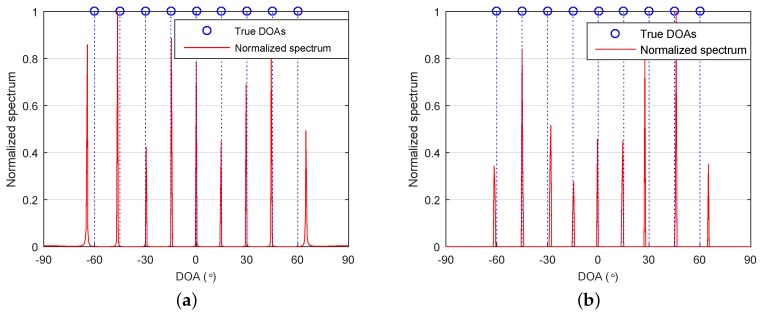
Normalized spectra of (**a**) the proposed method and (**b**) 4-L1. S={1,2,6}×d, K=9, $L=10^{4}$, signal–noise ratio (SNR)=20, and $r=0.5^{\circ }$. DOA: direction of arrival.

Consider an SLA with three sensors located at S={1,2,6}×d. It can be deduced that the associated FODCA has 19 distinct elements. That is, |D|=19, which is equal to sup{H} when M=3. According to Theorem 1, any greater than nine sources cannot be uniquely identified for the proposed method. Let us consider nine sources with DOAs $\left\{-60^{\circ },-45^{\circ },-30^{\circ },-15^{\circ },0^{\circ },15^{\circ },30^{\circ },45^{\circ },60^{\circ }\right\}$. The number of snapshots and SNR were set as $L=10^{4}$ and SNR=20 dB, respectively. Grid resolution was set to $r=0.5^{\circ }$. Figure 1a,b shows the normalized spectra of the proposed method and 4-L1, respectively. As can be seen, all DOAs of the sources were identified successfully. Note that this was unachievable for A-4-MUSIC and A-4-SS-MUSIC. The virtual array of A-4-MUSIC contained seven distinct virtual sensors, which could identify six sources at most. The largest ULA segment of the FOCDA contained 13 distinct elements, giving rise to a maximum identifiable number of six for A-4-SS-MUSIC.

### 4.2. Impact of the Grid Resolution

Consider an SLA {1,2,4,11,25}×d, which is constructed from an expanding and shift scheme that consists of two nested arrays {1,2,4}×d and {1,2,4}×d [18]. It can be obtained that the number of distinct elements of the corresponding FODCA and associated largest ULA segment is |D|=85 and ${\left|D\right|}_{\mathrm{ULA}}=55$, respectively. Consider two independent sources with DOAs $\left\{0^{\circ },20^{\circ }\right\}+\zeta_{1}$, where $\zeta_{1}$ is a random variable chosen uniformly within $\left[-1^{\circ },1^{\circ }\right]$. SNR was set at SNR=3 dB.

**Figure 2:**
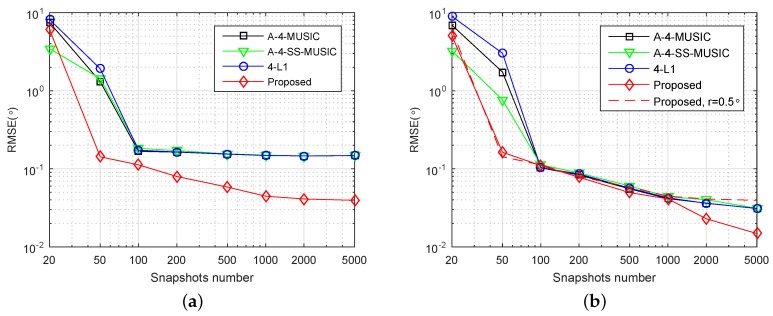
Root mean square error (RMSE) performance of four methods versus number of snapshots. S={1,2,4,11,25}×d, K=2, SNR=3 dB, (**a**) $r=0.5^{\circ }$ and (**b**) $r=0.1^{\circ }$.

Figure 2a,b shows the RMSE performance of the four methods with respect to number of snapshots, with grid resolution $r=0.5^{\circ }$ and $r=0.1^{\circ }$, respectively. In Figure 2a, when snapshots number was large, the RMSEs of A-4-MUSIC, A-4-SS-MUSIC, and 4-L1 did not reduce as snapshots increased, mainly because of the mismatch errors between grid and true DOAs. Due to the off-grid parameters estimation, the proposed method outperformed the other three methods for most of simulated snapshot numbers. In Figure 2b, the grid resolution is $r=0.1^{\circ }$, and it is clear that the RMSEs of A-4-MUSIC, A-4-SS-MUSIC, and 4-L1 increased to the same level of the proposed method when snapshots were greater than 50 and less than 2000. Nonetheless, the proposed method still outperformed the other three methods when snapshots were more than or equal to 2000. It can also be observed that the proposed method had smaller RMSE than A-4-MUSIC and 4-L1 when snapshots were less than 100. The RMSEs of A-4-SS-MUSIC were slightly larger than the other three methods when snapshots were greater than 50 and less than 5000, which may be caused by the fact that 4-SS-MUSIC does not utilize all the virtual sensors in associated FODCA. In addition, the dashed line in Figure 2b gives the RMSEs of the proposed method for $r=0.5^{\circ }$, and it is clear that the grid resolution seldom affected the RMSEs of the proposed method when snapshots were less than 2000, which implies that when snapshots are not too large, the proposed method can reduce the computational complexity by selecting a relatively coarser grid without heavy loss of RMSE performance.

### 4.3. Identifiability for Adjacent Sources

**Figure 3:**
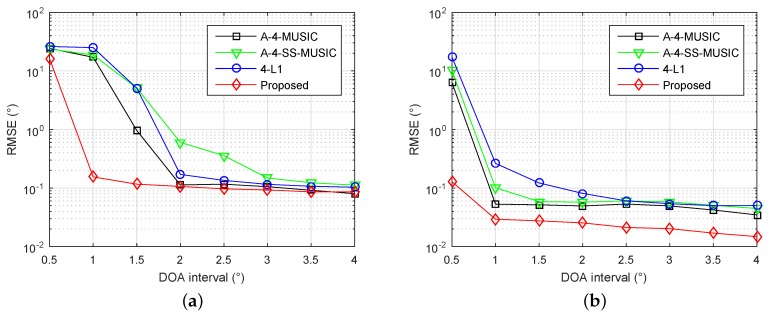
RMSE performance of four methods with different DOA intervals, S={1,2,4,11,25}×d, K=2, $r=0.1^{\circ }$, (**a**) L=300 and SNR=3 dB, (**b**) L=1000 and SNR=10 dB.

Next, we investigated the identifiability of adjacent sources using the proposed method. The array and parameters were set as in Section 4.2, except the grid resolution was set to $r=0.1^{\circ }$, and the DOAs of sources were set to $\left\{\zeta_{2},\zeta_{2}+▵\theta \right\}$, where $\zeta_{2}$ is a random variable chosen uniformly within $\left[-1^{\circ },1^{\circ }\right]$, and $▵\theta =0.5^{\circ },1^{\circ },\ldots ,5^{\circ }$ are the different DOA intervals. Figure 3a,b shows the RMSE performance of four methods with different DOA intervals, when the number of snapshots is 300 and SNR is 3 dB, and when the number of snapshots is 1000 and SNR is 10 dB, respectively. It is clear that the proposed method had smallest RMSEs for most simulated DOA intervals in both simulations, demonstrating the superior identifiability of adjacent sources with the proposed method.

### 4.4. Impact of SNR

**Figure 4:**
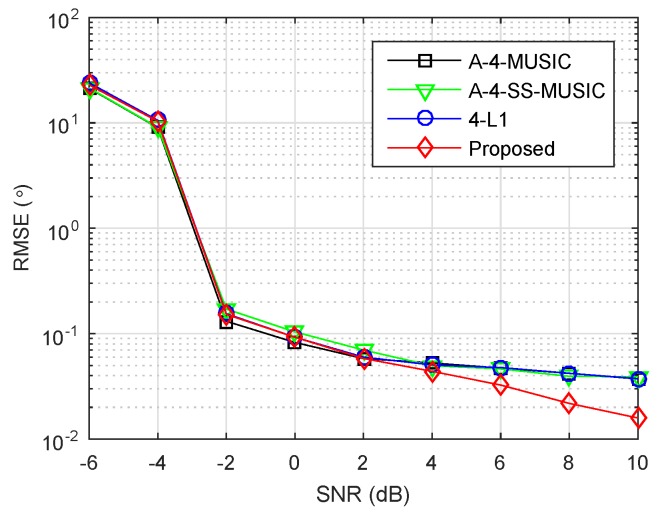
RMSE performance of four methods versus SNR, S={1,2,4,11,25}×d, K=2, $r=0.1^{\circ }$, L=500.

The array and parameters were set as in Section 4.2, except the grid resolution was set to $r=0.1^{\circ }$. Figure 4 plots the RMSEs of four methods as a function of SNR, where the number of snapshots was set to L=500. It is clear that the four methods had almost equal RMSEs when SNR was smaller than 4 dB. In the high SNR region, a relatively better performance of the proposed method appeared, benefiting from the off-grid parameter estimation as discussed in the large snapshot number region in Section 4.2.

### 4.5. Impact of Modelling Errors

**Figure 5:**
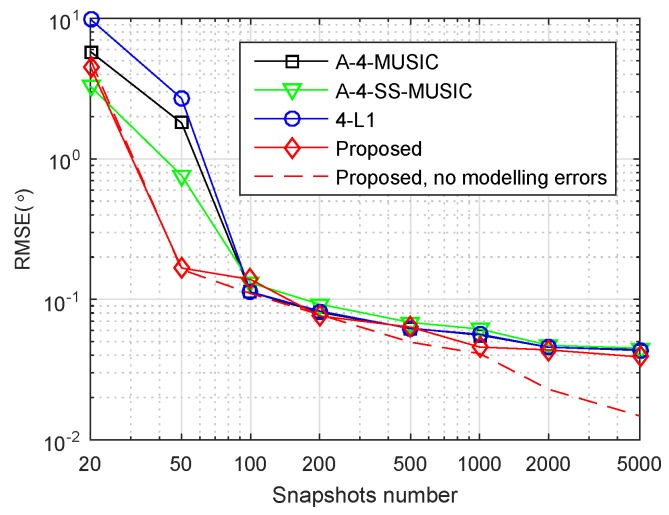
RMSE performance of four methods with modelling errors, S={1,2,4,11,25}×d, K=2, $r=0.1^{\circ }$, SNR=3 dB.

We also studied the effect of modelling errors on the proposed method. We used the same array geometry and parameters, and the same way of generating K=2 DOAs as in Section 4.4. We perturbed the steering vector by adding a modelling error vector $e_{a(\theta_{k})}$ to get the perturbed steering vector as $\tilde{a}\left(\theta_{k}\right)=a\left(\theta_{k}\right)+e_{a(\theta_{k})}$, where k=1,…,K, and $e_{a(\theta_{k})}$ are assumed to be zero mean statistically independent Gaussian distributed with $E\{e_{a(\theta_{k})}e_{a(\theta_{k})}^{H}=\sigma_{a(\theta_{k})}^{2}I$, where $\sigma_{a(\theta_{k})}^{2}=0.0025$. Figure 5 shows the RMSE performance of four methods with such modelling errors. In order to facilitate comparison, the RMSEs of the proposed methods with no modelling errors are also plotted in a dashed line. It can be observed that the RMSE performance of the four methods did not degrade heavily. However, when snapshots were more than 1000, the superior performance of the proposed method disappeared, suggesting that modelling errors did have a large impact on the off-grid parameters estimation of the proposed method.

### 4.6. Underdetermined Case

**Figure 6:**
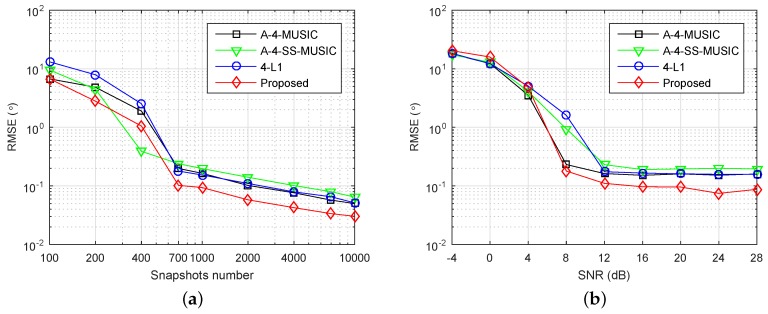
RMSE performance of four methods in the underdetermined case, S={1,2,4,11,25}×d, K=6, $r=0.1^{\circ }$, (**a**) SNR=20 dB, (**b**) snapshots number is L=1000.

We investigated the performance of the proposed method in underdetermined cases. The same array {1,2,4,11,25}×d was used, and the grid resolution $r=0.1^{\circ }$, while six independent sources with DOAs $\left\{-45^{\circ },-30^{\circ },-15^{\circ },0^{\circ },25^{\circ },40^{\circ }\right\}$ were set. Figure 6 shows the RMSE performance of the four methods. In Figure 6a, the number of snapshots varying from 100 to 10,000 and the SNR was fixed at 20 dB. It is clear that the proposed method had the smallest RMSEs for most of the simulated snapshot numbers. In Figure 6b, SNR varied from −4 dB to 28 dB and the snapshots number was fixed at 1000. It can be observed that the proposed method had smaller RMSEs when SNR>4 dB, but had slightly larger values when SNR≤4 dB, when compared with the other three methods. The RMSE performance of A-4-SS-MUSIC was obviously outperformed by the other three methods in the large snapshot number and high SNR regions, because 4-SS-MUSIC does not utilize all the virtual sensors in the associated FODCA. Moreover, the RMSEs of each method seemed to converge to a positive constant as SNR increased. A similar phenomenon can be observed and explained in covariance vector-based methods in the underdetermined case [22,26].

### 4.7. Non-Linear Array Case

**Figure 7:**
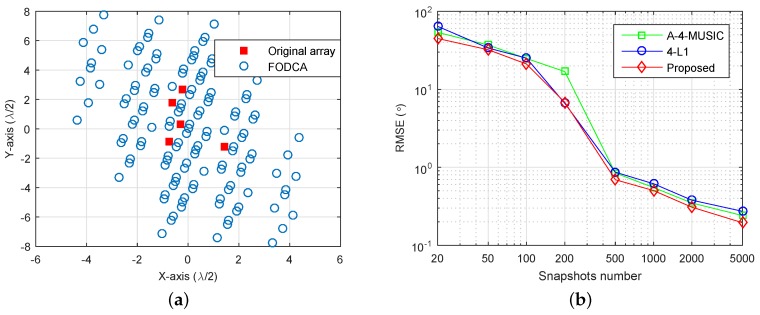
RMSE performance of three methods in the non-linear array case. M=5, K=3, $r=0.1^{\circ }$, (**a**) the array geometry and the corresponding FODCA geometry, (**b**) RMSE versus the number of snapshots, SNR=6 dB.

We now give an example to show the effectiveness of the proposed method in the non-linear array case. We randomly generated a planar array as shown in Figure 7a, where the minimum distance between sensors was half of the wavelength. The corresponding FODCA geometry is also illustrated, which had |D|=131 distinct elements. Note that the A-4-SS-MUSIC cannot be applied for the non-linear array case. We set three sources with DOAs $\left\{-60^{\circ },0^{\circ },40^{\circ }\right\}+\zeta_{3}$, where $\zeta_{3}$ is a random variable chosen uniformly within $\left[-1^{\circ },1^{\circ }\right]$. SNR was set at SNR=6 dB, and the RMSEs of A-4-MUSIC, 4-L1, and the proposed method were plotted as a function of the number of snapshots (Figure 7b). It can also be seen that the proposed method had better RMSE performance when snapshots were greater than 200.

### 4.8. Effectiveness for Non-Circular Sources

**Figure 8:**
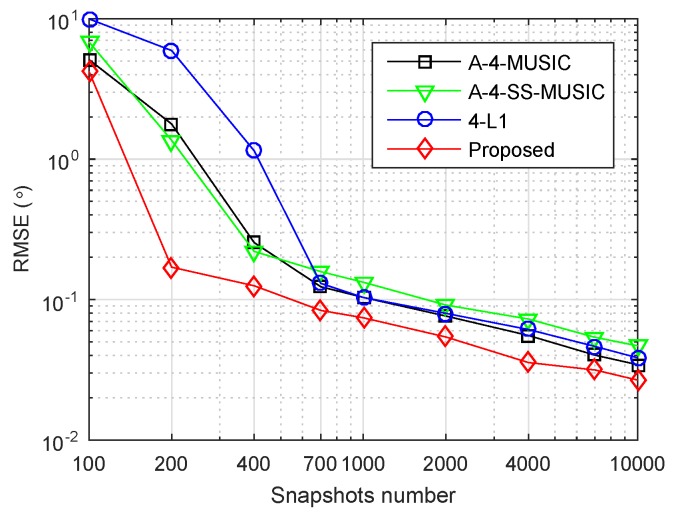
RMSE performance of four methods with non-circular sources, M=5, K=6, $r=0.1^{\circ }$, SNR=6 dB.

Finally, we consider the case of non-circular sources, using the same array {1,2,4,11,25}×d, and six independent sources with DOAs $\left\{-45^{\circ },-30^{\circ },-15^{\circ },0^{\circ },25^{\circ },40^{\circ }\right\}$ as in Section 4.6. In contrast, we replaced the imaginary parts of sources by their real parts. That is, $s_{\mathrm{NC}}\left(t\right)=R\left\{s\left(t\right)\right\}+jR\left\{s\left(t\right)\right\}$, where $s_{\mathrm{NC}}\left(t\right)$ is the non-circular sources to be simulated. We set SNR=20 dB and $r=0.1^{\circ }$. We estimated the FOCs by (19) for all the methods, and estimated $\hat{Q}$ by (27) for the proposed method. Figure 8 shows the RMSEs of the four methods versus the number of snapshots. Similar to Figure 6a, it is clear that the proposed method had the smallest RMSEs for all simulated snapshot numbers. Beyond doubt, all the methods were effective for non-circular sources with the estimation of FOCs by (19).

## 5. Conclusion

In this paper, a novel SMV sparse model for non-Gaussian sources using denoised FOCs vector is established to perform DOA estimation, and solved efficiently by the OGSBI method. An advanced denoising and dimension reduction procedure of FOCs vector is provided. The estimation errors of FOCs are integrated in the proposed SMV model, and are approximately estimated in a simple way. The proposed method suits any geometry, does not need prior knowledge of the number of sources, has maximum identifiability $O(M^{4})$, and is insensitive to associated parameters. The off-grid parameter estimation in our method promotes superior performance when the number of snapshots is large and SNR is high, and also ensures good performance when choosing a relatively coarse grid. Numerical simulations illustrate that the proposed method has superior identifiability or RMSE performance in different scenarios, namely grid resolution, adjacent sources, SNR, modelling errors, geometry, and non-circular sources, when compared with other state-of-the-art methods.

## Figures and Tables

**Table 1:** Comparison with state-of-the-art methods. 4-L1: fourth-order cumulants (FOCs) vector-based L1-norm method; A-4-MUSIC: non-redundant version of 4-MUSIC with averaging strategy; A-4-SS-MUSIC: FOCs vector-based spatial smoothing (SS)-MUSIC with averaging strategy; FODCA: fourth-order difference co-array; ULA: uniform linear array.

	A-4-MUSIC	A-4-SS-MUSIC	4-L1	Proposed
**Required geometry**	Any geometry	LA, with FODCA- containing ULA segment	Any geometry	Any geometry
**Maximum** **identifiability**	$O(M^{2})$	$O(M^{4})$	$O(M^{4})$	$O(M^{4})$
**Prior knowledge** **of the number of sources**	Need	Need	No need	No need
**Handles** **correlated sources**	Yes	No	No	No
**Parameters and** **sensitivity**	With no parameters	With no parameters	With sensitive parameters	With insensitive parameters
**Complexity**	$O(LM^{4}+IM^{4}+M^{6})$	$O(LM^{4}+IM^{8}+M^{12})$	$O(LM^{4}+I^{3}T_{2})$	$O(LM^{8}+I^{2}M^{4}T_{1})$

## Appendix A. The Proof of Property 1

**Proof.** Consider the linear equation $\sum_{\xi =1}^{\left|D\right|}\upsilon_{\xi }J_{\xi }=0$, where $J_{\xi }$ is the ξth column vector of J. For any $n_{i_{1}},n_{i_{2}},n_{i_{3}},n_{i_{4}}\in S$, it can be deduced that $0={\left[\sum_{\xi =1}^{\left|D\right|}\upsilon_{\xi }J_{\xi }\right]}_{i}=\sum_{\xi =1}^{\left|D\right|}\upsilon_{\xi }J_{i,\xi }=\upsilon_{\xi_{0}}$, where the index $\xi_{0}$ satisfies that associated vector $m_{\xi_{0}}=n_{i_{1}}+n_{i_{2}}-n_{i_{3}}-n_{i_{4}}$. Due to the arbitrariness of $n_{i_{1}},n_{i_{2}},n_{i_{3}},n_{i_{4}}\in S$, the coefficients $\upsilon_{\xi }=0$ for all $\upsilon_{\xi }=1,2,\ldots ,\left|D\right|$, implying that J has full column rank. The proof is completed. ☐

## Appendix B. The Proof of Property 2

**Proof.** It is obvious that $Ja_{D}\left(\theta \right)=\sum_{\xi =1}^{\left|D\right|}J_{\xi }e^{j2\pi u^{T}m_{\xi }}$. According to Definition 2, we can obtain ${\left[\sum_{\xi =1}^{\left|D\right|}J_{\xi }e^{j2\pi u^{T}m_{\xi }}\right]}_{i}=e^{j2\pi u^{T}\left(n_{i_{1}}+n_{i_{2}}-n_{i_{3}}-n_{i_{4}}\right)}$. On the other hand, the *i*th row of b(θ) is ${\left[b\left(\theta \right)\right]}_{i}=a_{i_{1}}\left(\theta \right)a_{i_{2}}\left(\theta \right)a_{i_{3}}^{*}\left(\theta \right)a_{i_{4}}^{*}\left(\theta \right)=e^{j2\pi u^{T}\left(n_{i_{1}}+n_{i_{2}}-n_{i_{3}}-n_{i_{4}}\right)}$. Thus, we have $b\left(\theta \right)={\mathrm{Ja}}_{D}\left(\theta \right)$. The proof is completed. ☐

## Appendix C. The Proof of Property 3

**Proof.** Consider Definition 1. For groups $\left\{n_{i_{1}},n_{i_{2}}\right\}$ and $\left\{n_{i_{3}},n_{i_{4}}\right\}$, we have two cases as follows. Case 1: the same element does not exist between $\left\{n_{i_{1}},n_{i_{2}}\right\}$ and $\left\{n_{i_{3}},n_{i_{4}}\right\}$. If $n_{i_{1}}=n_{i_{2}}$ and $n_{i_{3}}=n_{i_{4}}$, there are (M1)(M−11) distinct elements at most; if $n_{i_{1}}=n_{i_{2}}$ and $n_{i_{3}}\neq n_{i_{4}}$, there are (M1)(M−12) distinct elements at most; if $n_{i_{1}}\neq n_{i_{2}}$ and $n_{i_{3}}=n_{i_{4}}$, there are (M2)(M−21) distinct elements at most; if $n_{i_{1}}\neq n_{i_{2}}$ and $n_{i_{3}}\neq n_{i_{4}}$, there are (M2)(M−22) distinct elements at most. Case 2: the same element exists between $\left\{n_{i_{1}},n_{i_{2}}\right\}$ and $\left\{n_{i_{3}},n_{i_{4}}\right\}$. Without loss of generality, assuming $n_{i_{2}}=n_{i_{4}}$, if $n_{i_{1}}=n_{i_{3}}$, there is one element 0; if $n_{i_{1}}\neq n_{i_{3}}$, there are M(M−1) distinct elements at most. If there is no other redundancy, the max |D| is obtained by summing over all the cases, which is

(A1) $$\begin{array}{c} {\left|D\right|}_{\max}\left(M\right)=\left(M^{4}-2M^{3}+7M^{2}-6M+4\right)/4. \end{array}$$

Next, we provide an example to show that the maximum ${\left|D\right|}_{\max}\left(M\right)$ can be achieved for any *M*. Consider the linear array $S=\{10^{0},10^{1},\ldots ,10^{M-1}\}$. It is not hard to find that in both of the above cases all the maxima can be achieved due to the different orders of magnitude of the elements in S. Thus, ${\left|D\right|}_{\max}\left(M\right)$ can be achieved for any *M*. Therefore, combining the definition of H, it is clear that the supremum of H is $\sup\left\{H\right\}=\left(M^{4}-2M^{3}+7M^{2}-6M+4\right)/4$ for given *M*. The proof is completed. ☐

## Appendix D. The Proof of Lemma 1

**Proof.** It is clear that $\mathrm{var}\left(\bar{Y_{t}Z_{t}}\right)=\mathrm{var}\left(\sum_{t=1}^{L}Y_{t}Z_{t}/L\right)=\mathrm{var}\left(Y_{t}Z_{t}\right)/L$, and $\mathrm{var}\left({\bar{Y}}_{t}{\bar{Z}}_{t}\right)=\mathrm{var}\left(\sum_{t_{1}=1}^{L}Y_{t_{1}}\cdot \sum_{t_{2}=1}^{L}Z_{t_{2}}/L^{2}\right)=\mathrm{var}\left(Y_{t}Z_{t}\right)/L^{3}+\mathrm{var}\left(Y_{t}\right)\mathrm{var}\left(Z_{t}\right)\left(L-1\right)/L^{3}$. Since $\mathrm{var}(Y_{t}Z_{t})$ and $\mathrm{var}\left(Y_{t}\right)\mathrm{var}\left(Z_{t}\right)$ are independent from *L*, it can be deduced that as *L* approaches to infinity, $\mathrm{var}\left(\bar{Y_{t}Z_{t}}\right)/\mathrm{var}\left({\bar{Y}}_{t}{\bar{Z}}_{t}\right)=O\left(L\right)$. The proof is completed. ☐

## Appendix E. The Proof of Theorem 1

**Proof.** Due to Proposition 1, if any *K* sources can be uniquely identified from model (8), we have $K<\mathrm{spark}\{B_{\theta }\}/2$. From (46), we obtain K<(|D|+1)/2. Applying Property 3, we can obtain $K<(M^{4}-2M^{3}+7M^{2}-6M)/8+1$. As *K* is an integer, it is obvious $K\leq (M^{4}-2M^{3}+7M^{2}-6M)/8$. The proof is completed. ☐
